# Hysterosalpingographic Appearances of Female Genital Tract Tuberculosis: Part II: Uterus 

**Published:** 2014-03-09

**Authors:** Firoozeh Ahmadi, Fatemeh Zafarani, Gholam Shahrzad Shahrzad

**Affiliations:** Department of Reproductive Imaging at Reproductive Biomedicine Research Center, Royan Institute for Reproductive Biomedicine, ACECR, Tehran, Iran

**Keywords:** Female Genital Tuberculosis, Hysterosalpingography, Endometrium

## Abstract

Female genital tuberculosis remains as a major cause of tubal obstruction leading to infertility, especially in developing countries. The global prevalence of genital tuberculosis has increased during the past two decades due to increasing acquired immunodeficiency syndrome. Genital tuberculosis (TB) is commonly asymptomatic and it is diagnosed during infertility investigations. Despite of recent advances in imaging tools such as computed tomography (CT) scan, magnetic resonance imaging (MRI) and ultrasongraphy, hysterosalpinography has been considered as the standard screening test for evaluation of tubal infertility and as a valuable tool for diagnosis of female genital tuberculosis. Tuberculosis gives rise to various appearances on hysterosalpingography (HSG) from non-specific changes to specific findings. The present pictorial review illustrates and describes specific and non-specific radiographic features of female genital tuberculosis in two parts. Part I presents specific findings of tuberculosis related to tubes such as "beaded tube", "golf club tube", "pipestem tube", "cobble stone tube" and the "leopard skin tube". Part II will describe adverse effects of tuberculosis on structure of endometrium and radiological specific findings, such as "T-shaped" tuberculosis uterus, "pseudo-unicornuate "uterus, "collar-stud abscess" and "dwarfed" uterus with lymphatic intravasation and occluded tubes which have not been encountered in the majority of non-tuberculosis cases.

## Introduction

Female genital tuberculosis (FGTB) is one form of extrapulmonary manifestations of tuberculosis and includes 5% of all female pelvic infections (1, 2). It is more frequent in developing countries, leading to chronic pelvic inflammatory disease (PID) and infertility (3).

The reported prevalence of genital tuberculosis has shown a descending trend in developed countries, but recently, its rate has started to increase again due to co-infection with human immunodeficiency virus (HIV) and the development of drug resistants trains of Mycobacterium tuberculosis (4-6). Primary infection of the female genital organs is very rare (7), and is secondary to a tuberculosis infection elsewhere in the body, usually the lungs (8, 9).

Diagnosis of genital TB may be difficult because majority of cases are asymptomatic; furthermore, in high prevalence-countries, culture facilities for mycobacterium and histopathologic diagnosisare limited (9-11). In these circumstances, the infection has been usually diagnosed during hysterosalpingography for preliminary investigations of infertility (12, 13). In addition, hysterosalpingography is still the golden standard method for evaluation of tubal lumen (14), and a helpful procedure in diagnosis of female genital tuberculosis (15, 16). Tuberculosis gives rise to various appearances on hysterosalpingography (HSG) from non-specific changes to specific findings.

This part of pictorial review illustrates and describes endometrial changes following genital tuberculosis detected by HSG. 

### Pathologyand clinical presentation of endometrial tuberculosis

Tubal tuberculosis is disseminated to the endometrium in approximately 50% of cases (8) and persists in the basal layer, whereas is not shed during menstruation, or becomes re-infected from the tubes following menstruation. Thus, tubercles in the endometrium are always young. Tuberculous uterus may show a range of mild to severe endometritis including epitheliod granulomas with sparse, endometrial ulcer leading to partial or complete intrauterine adhesions, obliteration and deformity of the uterus. The involvement of myiometrium is seen in 2.5% of patients with abscess formation (2).

The pathognomonic findings for tuberculosisinclude specific and non-specific features. Specific radiographic features are "collar-stud abscess", "T-shaped" uterus and unicornuate uterus-likeappearance (the "pseudounicornuate" uterus). Other uterine changes due to tuberculosis known as nonspecific features include endometritis, synechiae, distortion of uterine contour, and venous and lymphatic intravasations (14, 15).

### Intrauterine adhesions and distortion

**Fig 1:**
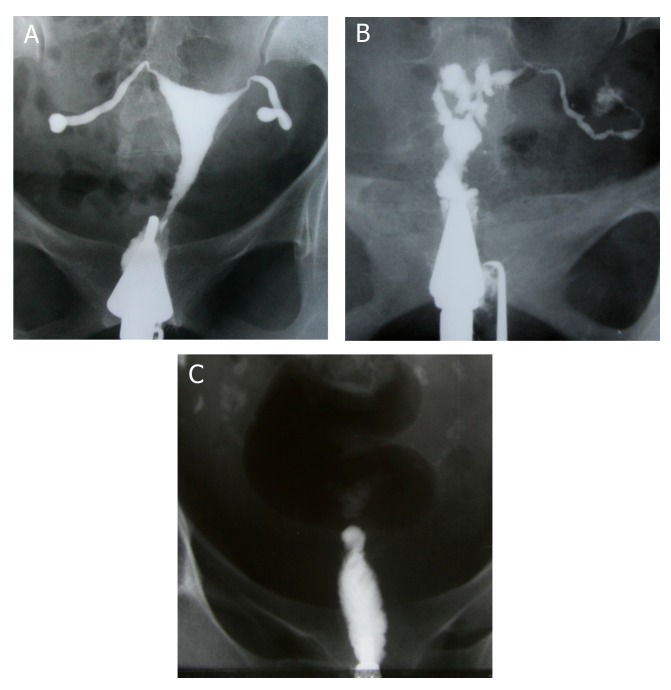
A. Hysterosalpingographic appearance of genital TB in a patient with primary infertility. Uterine cavity is normal in shape and size. Terminal sacculation are seen in both tubes. B. Irregularity, multiple filling defects and obliteration of right ostium secondary to extensive synechiae formation in this site. Obstruction of left tube is also seen. C. Complete obliteration of uterine cavity due to extensive synechiae formation following chronic uterine TB.

Uterine manifestations in tuberculosis may vary from mild endometritis to severe scarring and deformity leading to total obliteration of the uterine cavity (Fig 1A-C) (16). In mild endometritis, the endometrial involvement is usually superficial, while uterine cavity has normal size, shape, and tonicity, whereas there is always obstruction of both tubes.

Later with progression of TB, caseation and ulceration of endometrium occur, and intrauterine scarring may result in synechiae and intrauterine adhesions. In this stage, the uterine cavity is usually normal in size, but irregularity of uterine contour, filling defects, lack of uterine contractility and tubal patency may be seen (17).

**Fig 2:**
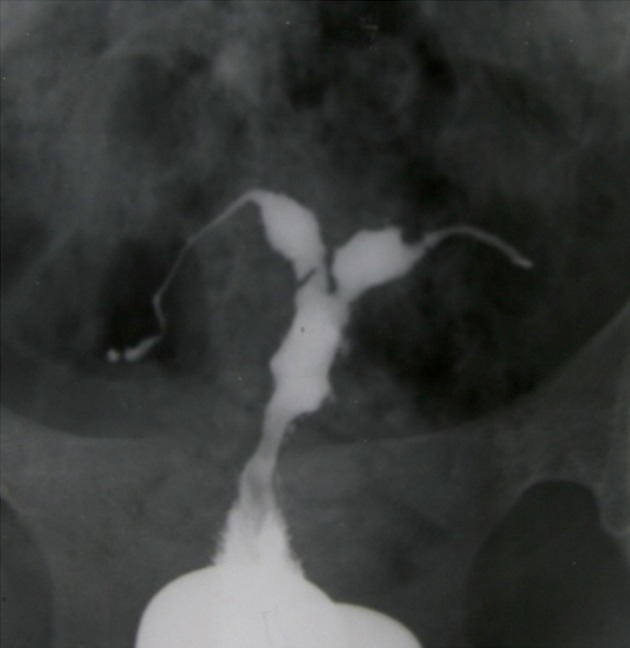
Indentation of the cavity due to synechiae resembles a denticulate uterus .Obstruction of the isthmic portion in both tubes is present.

**Fig 3:**
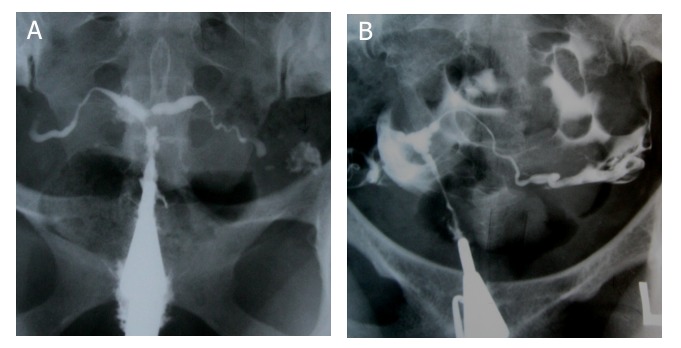
T-shaped configuration in two different patients. A. "T-shaped" tuberculosis uterus. Irregular contour of the uterine cavity with diminished capacity resembling a T-shaped uterus. Both tubes are obstructed from isthmic portion. B. T-shaped uterus due to DES exposure. Narrow endocervical canal and small uterine cavity. Note both tubes are normal.

**Fig 4:**
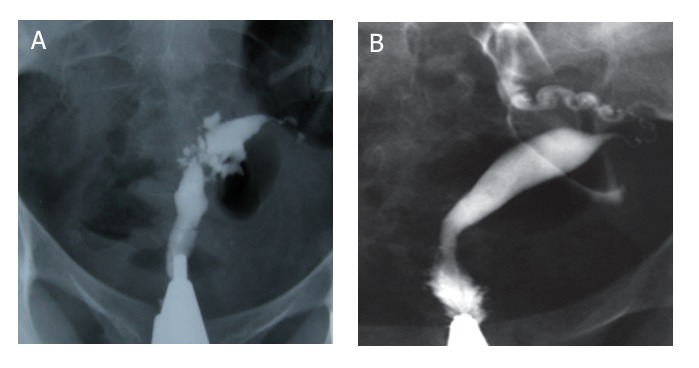
A. Pseudo-unicornuate uterus. Unilateral scarring of the cavity makes an asymmetric intrauterine obliteration, resembling a unicornuate uterus. Note the irregular contour and vertical orientation of long axis. B. True unicornuate uterus. Note the smooth contour, more horizontal orientation of long axis and normal ipsilateral fallopian tube.

With progression of disease, irregularity of uterine contour and filling defects may result in a denticulate cavity (Fig 2) (18, 19), or may convert the triangular uterine cavity into a T- shape which is very similar to diethylstilbestrol uteri (14, 19). A "T-shaped" tuberculosis uterus should be differentiated from a "T-shaped" in diethylstilbestrol (DES) exposure. The characteristic appearances of DES uterus are usually T-shaped with multiple constriction bands, a boxlike lower uterine cavity, a narrow endocervical canal and a hypoplastic uterine cavity; however, the fallopian tubes are usually normal (Fig 3) (20). Sometimes, unilateral scarring of the cavity results in obliteration of cavity on one side of uterus giving rise to a unicornuateappearance called a "pseudounicornuate" uterus (19). True unicornuate uterus can be differentiated from a pseudo unicornuate uterus by having a smooth contour, a more horizontally oriented long axis and normal ipsilateral fallopian tube (Fig 4A, B) (14). 

**Fig 5:**
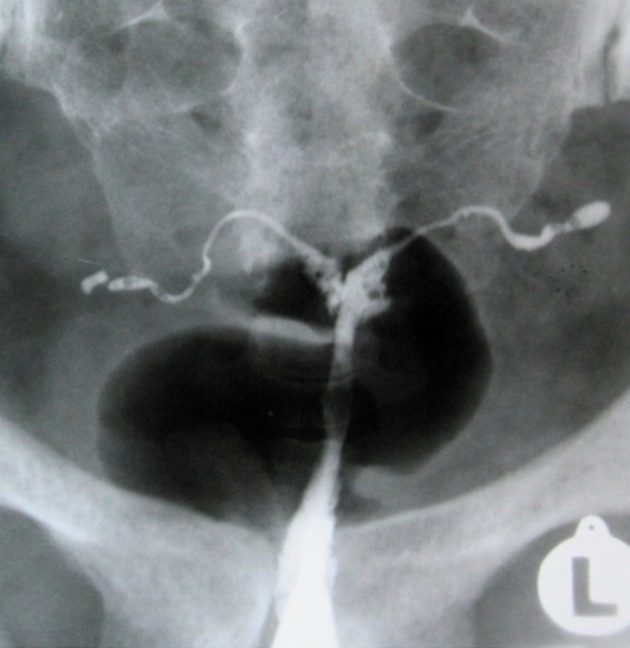
A. Dwarfed uterus. HSG shows a very small, shrived and deformed uterine cavity. Disproportion between uterine cavity and cervical canal is obvious. Both tubes are occluded

**Fig 6:**
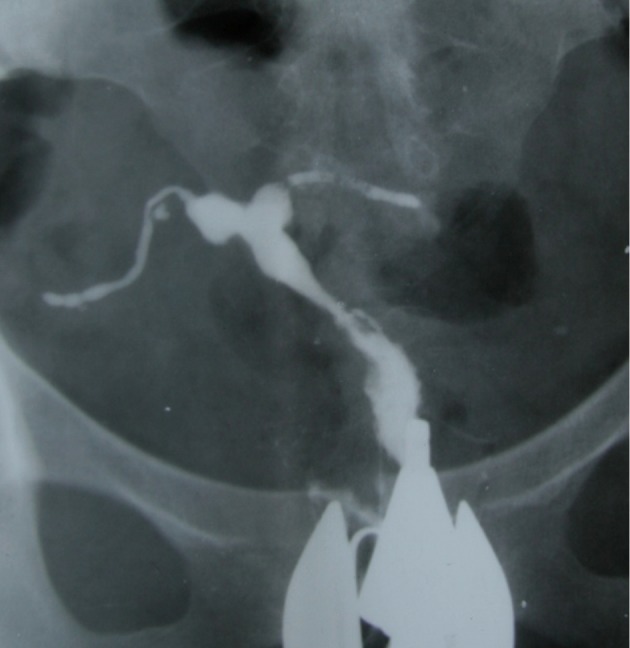
Trifoliate shaped uterus. Synechiae formation at the uterine borders and partial obliteration in the fundus produce a trifoliate like appearance. Both tubes are obstructed in the isthmic portion.

A dwarfed uterus which is characterized with a small and shrived uterus with irregularity and disproportion between uterine cavity and cervix, while trifoliate shaped uterus are other presentations of uterine tuberculosis (Figs 5, 6) (17).

**Fig 7:**
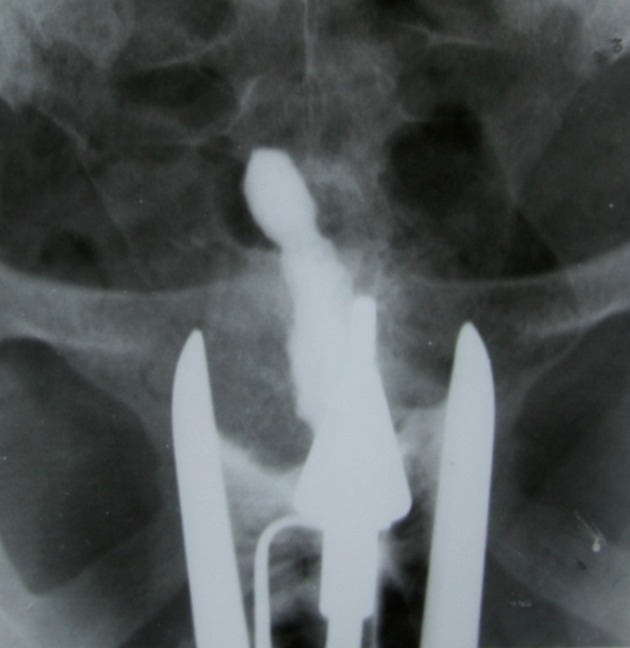
Netter syndrome. Obliteration of entire uterine cavity due to extensive synechiae formation giving of glove’s finger appearance.

After long duration of infection, extensive destruction of endometrium and myometrium followed by fibrosis and complete obliteration of the uterine cavity may occur as the "Netter syndrome" (21). Hysterosalpingographic characteristic of Netters syndrome is called "glove’s finger" consisted of a cervical canal and a small part of the uterus (Fig 7) (21). Other radiographic findings of tubercular affection of the uterus include the formation of a "collar-stud abscess", which is pathognomonic for tuberculosis (14). This feature should be differentiated from intracavitary changes due to necrosis in an adjacent uterine leiomyoma. A collar-stud abscess classically has a narrow neck with a broader base which is away from the endometrial cavity.

### Venous and lymphatic intravasation

The venous and lymphatic intravasation in uterine and adnexal vessels is acomplicated disorder which occurs due to progressive destruction and ulceration of endometrium.The most important cause of intravasation is the entry of contrast medium to the venous and lymphatic canals through unprotected ves- sels. Although this feature is not specific for tuberculosis, it can be detected by HSGs performed early in the menstrual cycle, shortly after endometrial instrumentation or pathological deficiency of endometrium (22). It is a good indicator for suggesting endometrial tuberculosis.

In hysterosalpingography filling of multiple, parallel beaded channels are seen.

**Fig 8:**
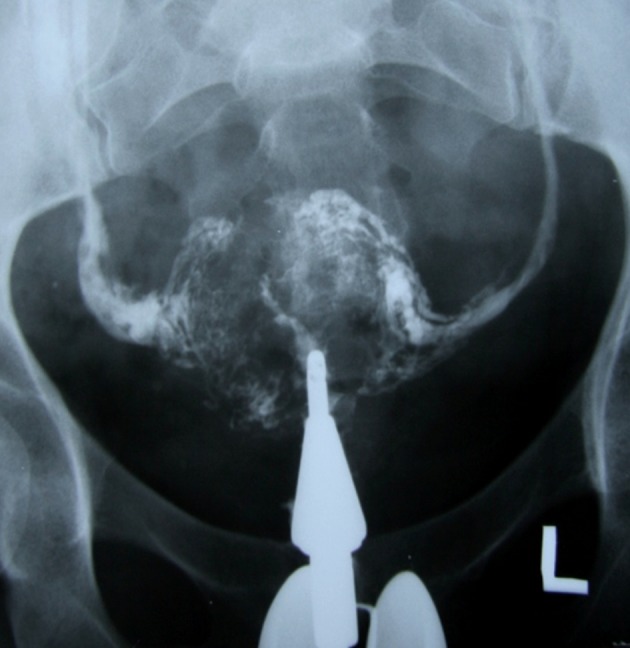
Complete obliteration of the uterine cavity. Extrauterine intravasation of contrast into the pelvic vein and lymphatic system is seen.

Contrast in thin delicate lymphatics are differentiated from blood vessels by their narrower caliber and reduced draining of contrast (Fig 8). 

### Cervical tuberculosis

 Cervical tuberculosis is rare due to the nature of stratified squamous epithelium of the ectocervix which causes to be resistance to bacterial penetration (23- 26). The disease is commonly secondary to tuberculous salpingitis and endometritis, while in primary form is usually transmitted by the partner with genitourinary TB (25, 26). TB cervix can coexist with carcinoma In-situ and infertility. Other common presentations are abnormal vaginal discharge/bleeding and menstrual irregularities (23, 24).

In the cervix, the tuberculous lesion can be ulcerative or proliferative. In the ulcerative form, the ulcers have wavy borders, clean cut edges and a yellow base. The proliferative lesion has papillary formations which may be pedunculated or sessile.

**Fig 9:**
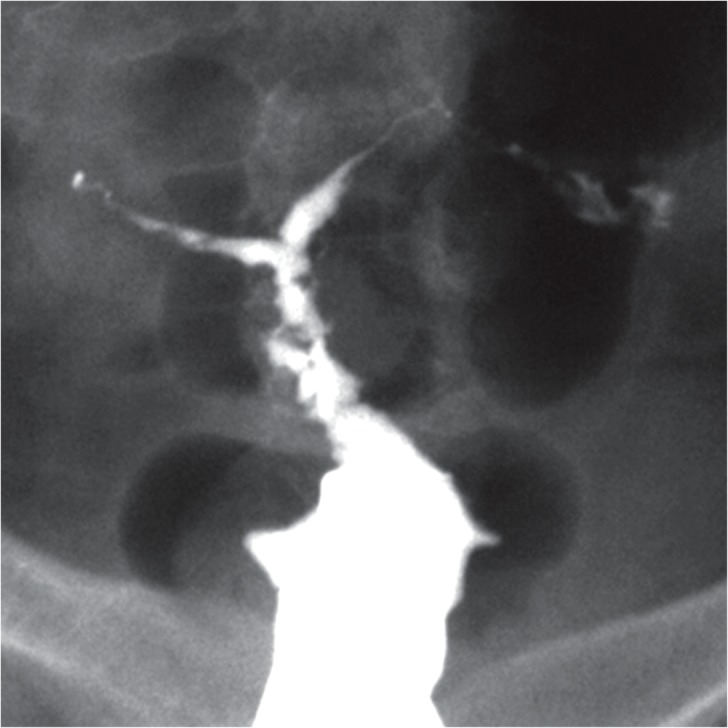
Cervical tuberculosis. Uterine cavity has small size, diverticular outpunching, ragged irregular contours and obvious deformity. Cervical filling defects, irregularity of cervical lumen and diverticular outpouchings are present. Occlusion of both tubes is also seen.

On HSG, caseous ulceration of the mucosa produces ragged irregular contours and diverticular outpouching with a feathery appearance (Fig 9). The other various features such as adhesions, distortion and a serrated endocervical canal have also seen in some cases.

### Reliable diagnostic criteria for female genital tuberculosis

**Fig 10:**
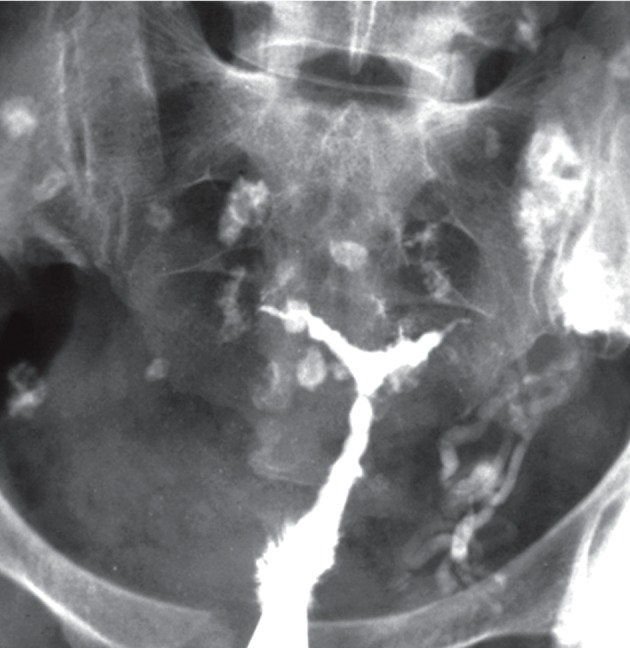
Pelvic tuberculosis in patient with chronic genital TB. Uterine cavity is small, deformed with irregular contour. Both tubes are occluded. Several calcified lymph nodes in the pelvis and intravasation of contrast into the veins are visualized (22).

There are useful differential diagnostic criteria suggested by Klein et al. (27) for diagnosis of tuberculosis as follows (Fig 10):

1. Calcified lymph nodes or smaller irregular calcifications in the adnexal area.
2. Obstruction of the fallopian tube in the zone of transition between the isthmus and the ampulla.
3. Multiple constrictions along the course of the fallopian tube.
4. Endometrial adhesion and/or deformity or obliteration of the endometrial cavity in the absence of curettage or of surgical termination of pregnancy.

## Conclusion

Uterine tuberculosis may show a range of mild to severe endometritis, restricted to superficial layers of endometrium or endometrial ulcer leading to progressive destruction, obliteration and deformity of the uterus in the late stages.

Some of the hysterosalpingographic findings of uterine tuberculosis, such as "T-shaped" tuberculosis uterus, "pseudounicornuate" uterus, "collarstud abscess" and "dwarfed" uterus with lymphatic intravasation and occluded tubes, are specific for female genital tuberculosis and have not been encountered in the majority of non-tuberculosis cases. Diagnosis of these radiographic characteristics is reliable evidence of genital tuberculosis and iscrucial in the infertility workup in order to make a proper decision. 
